# Novel Product for the Management of Coronary Ruptures Happening during Percutaneous Coronary Interventions

**DOI:** 10.1155/2021/6688338

**Published:** 2021-02-11

**Authors:** Arash Hashemi, Ashkan Hashemi, Arsis Ahmadieh, Azin Alizadehasl, Mohammad Mostafa Ansari Ramandi

**Affiliations:** ^1^Erfan General Hospital, Tehran, Iran; ^2^Weill Cornell Medicine, New York, New York, USA; ^3^Rajaie Cardiovascular, Medical and Research Center, Iran University of Medical Sciences, Tehran, Iran; ^4^Cardiovascular Diseases Research Center, Birjand University of Medical Sciences, Birjand, Iran; ^5^Network of Immunity in Infection, Malignancy and Autoimmunity (NIIMA), Universal Scientific Education and Research Network (USERN), Tehran, Iran

## Abstract

Coronary artery perforation during percutaneous coronary interventions is a rare but dreaded complication. One of the treatment methods for this complication is the injection of an obliterating material into the ruptured vessel. We will introduce a novel material named “Spongostan” for embolization with significant advantages over available treatment options.

## 1. Introduction

Coronary perforation is a potentially fatal complication of PCI [1]. It is a rare complication with more prevalence in females and patients with hypertension, previous coronary artery bypass grafting, and those admitted with NSTEMI [2]. It also has a higher incidence during complex procedures like bifurcation stenting, very tortuous or heavily calcified vessels, use of atheroablation devices, or CTO procedures [1, 2]. The most common cause of these perforations is wire tip induced [3]. This complication can be fatal and needs to be addressed properly. With more and more aggressive and advanced techniques or wires like heavy CTO-dedicated wires used, the rate of this complication rises [1–3]. Ellis classification (Table 1) shows the different types of coronary perforations [4].

Its incidence is between 0.35 and 0.52 percent [5]. A very high index of suspicion, timely diagnosis, and often definitive treatment are required to minimize adverse outcomes from this complication [5, 6].

## 2. Case Presentation

A 50-year-old female presented to our hospital with increased intensity of exertional retrosternal chest pain. She had exertional chest pain from a few days before and experienced a more severe form of pain from 3 hours before admission with slight exertion. The pain had a compressive nature without radiation and was accompanied by diaphoresis. On physical examination, she had a blood pressure of 135/85 mmHg, a heart rate of 86 beats/minute, and a respiratory rate of 20/minute. She had no fever. There was no remarkable finding in the physical examination.

### 2.1. Past Medical History

She had a history of controlled hypertension from 10 years before. She was a smoker for 15 years (7.5 pack-year). No history of cardiac disease or diabetes mellitus was present.

### 2.2. Differential Diagnosis

Considering her past medical history and sign and symptoms, acute coronary syndrome was the main diagnosis.

### 2.3. Investigations

Electrocardiography was done for the patient that revealed ST-segment depression in anterior and lateral precordial leads.

On laboratory testing, she had troponin I elevated to fivefolds of the normal upper limit.

**Figure 1:**
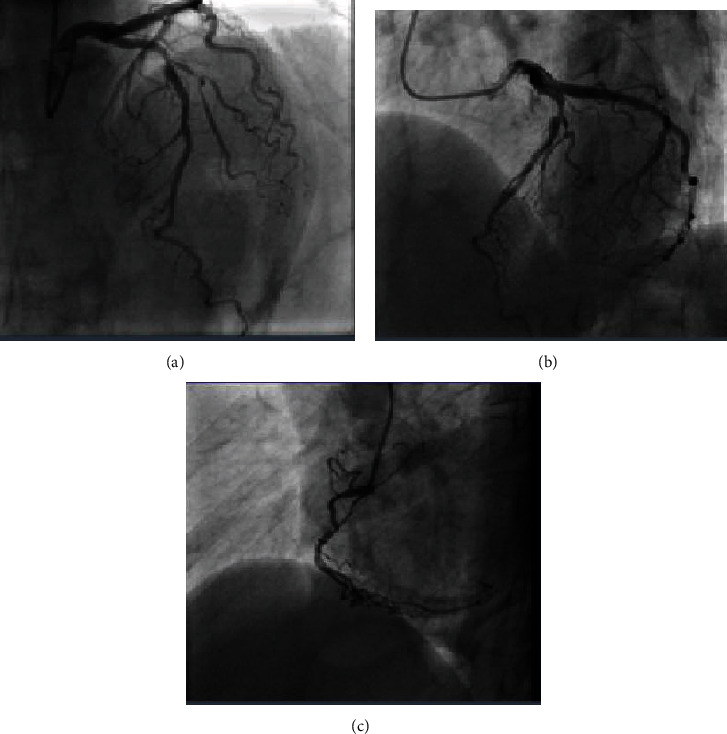
(a) Anterior-posterior (AP) cranial projection showing the stenosis in LAD-diagonal bifurcation. (b) Left anterior oblique (LAO) cranial projection showing the stenosis in LAD-diagonal bifurcation. (c) Right anterior oblique projection showing stenosis in RCA.

Considering the diagnosis of non-ST-segment elevation-myocardial infarction (NSTEMI) and due to the refractory angina after the event, she underwent diagnostic coronary angiography which revealed two-vessel diseases with significant stenosis in left anterior descending- (LAD-) diagonal bifurcation (Video 1) and proximal right coronary artery (RCA) and a low SYNTAX score (Figure 1).

Therefore, she was planned to undergo stage percutaneous coronary intervention (PCI) on LAD-diagonal bifurcation and then proximal RCA.

### 2.4. Management

**Figure 2:**
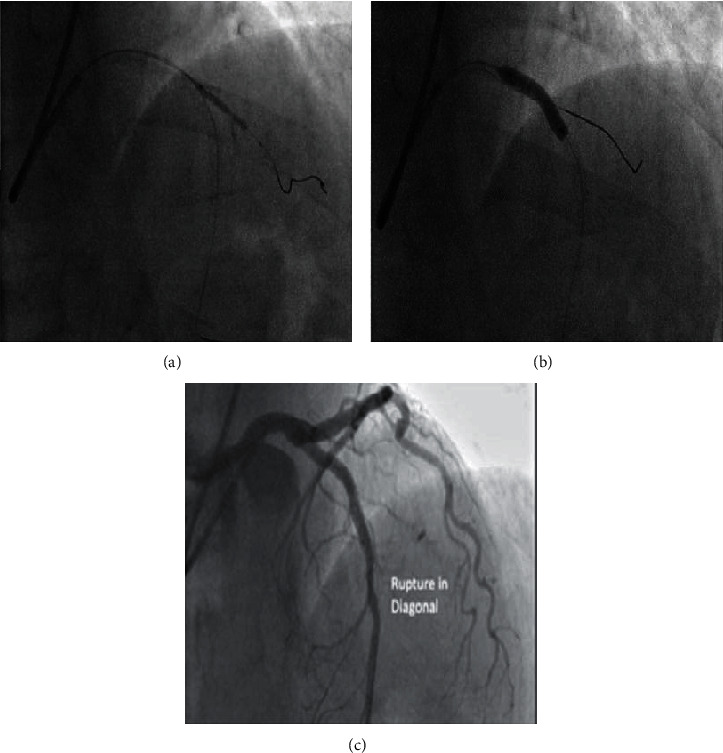
(a) Stenting in LAD and POT. (b) Rewiring in diagonal after its total occlusion. (c) Diagonal rupture.

Our plan for LAD-diagonal bifurcation was to perform provisional stenting and after wiring both branches' predilation in both vessels and finally, provisional stenting in LAD (Video 2) with postdilation, and final proximal optimization technique (POT) was done (Figure 2(a)). After final POT, the patient developed severe refractory chest pain and ST-segment changes due to closure of the jailed diagonal artery so we decided to perform rewiring (Video 3) and balloon opening of the occluded diagonal (Figure 2(b)). After rewiring and ballooning the diagonal, we noticed an iatrogenic rupture in the diagonal artery caused by the wire tip (Video 4, 5) (Figure 2(c)).

We started step-wise actions for treatment and management of this rupture including prolonged balloon inflation (Video 6) and serial echocardiographic evaluations. After three episodes of prolonged balloon inflations using a 2 × 20 mm balloon, it seemed that we managed to seal the perforation and there was no more leakage (Video 7). The echocardiographic evaluation showed mild to moderate pericardial effusion, and we terminated the procedure and sent the patient to the coronary care unit (CCU) and kept her under observation.

**Figure 3:**
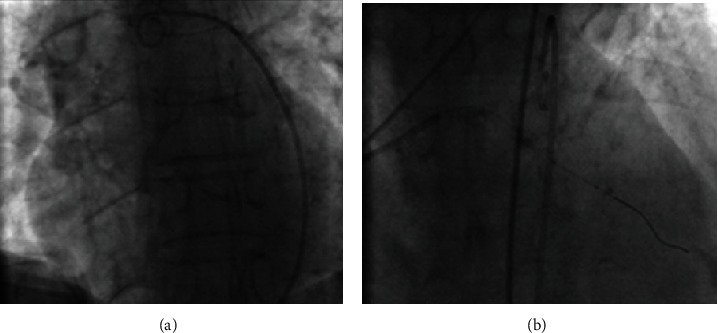
(a) Pigtail insertion. (b) Repeated wiring of the diagonal and prolonged balloon inflation.

Four hours later, she had low blood pressure and paradoxical pulse. On serial echocardiography, there were right atrium and right ventricle collapse in favor of tamponade. We quickly sent the patient to the catheterization laboratory. After drainage of the blood from the pericardium and pigtail insertion (Figure 3(a)), we reevaluated the ruptured vessel with angiography and found out that the leakage was persistent (Video 8). Repeated diagonal wiring and prolonged balloon inflation were done again with no luck (Video 9), and we decided to manage the rupture more aggressively (Figure 3(b)).

**Figure 4:**
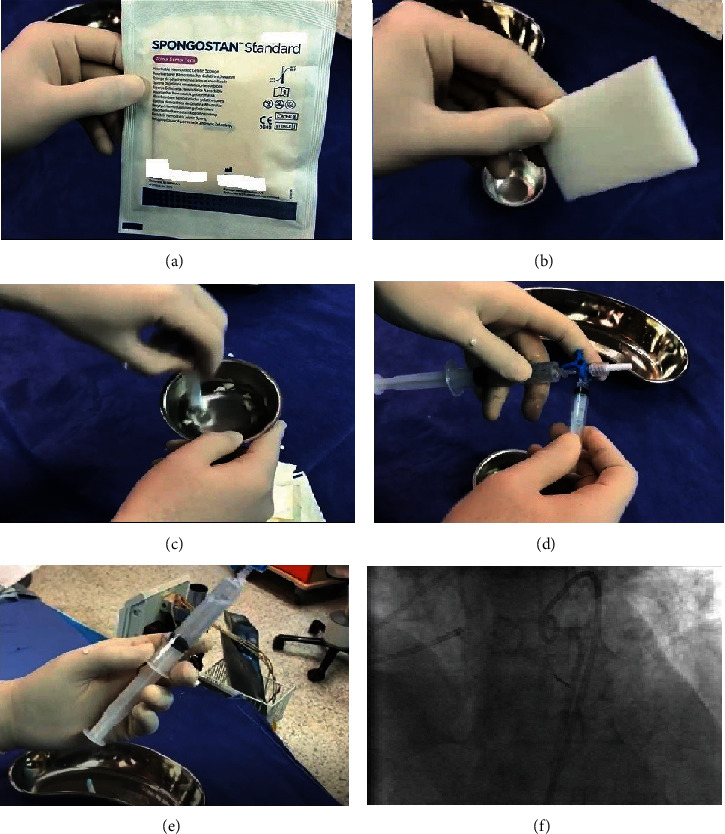
(a) Spongostan package. (b) Spongostan sponge. (c) Stirring and soaking one-fifth of the sponge in contrast media. (d) Dissolving the sponge using two syringes. (e) Dissolved Spongostan in the contrast. (f) Injection through microcatheter tip.

Our options at this point were to deploy a stent graft in the main LAD vessel or try to occlude the diagonal using coils or polyvinyl alcohol (PVA) or standard glue material. All these materials and methods have their costs and pitfalls so we decided to use a novel product for sealing the rupture. This product is called “Spongostan” and is a form of Gel foam commonly used by ear, nose, and throat (ENT) surgeons. It is very easy to use, and its preparation takes under two minutes. First, we dissolved the product with standard contrast, and then, the injection was made through a microcatheter tip (Figure 4).

After injection (Video 10), complete sealing of the ruptured vessel was achieved (Video 11). The patient was then sent to the CCU.

### 2.5. Follow-Up

**Figure 5:**
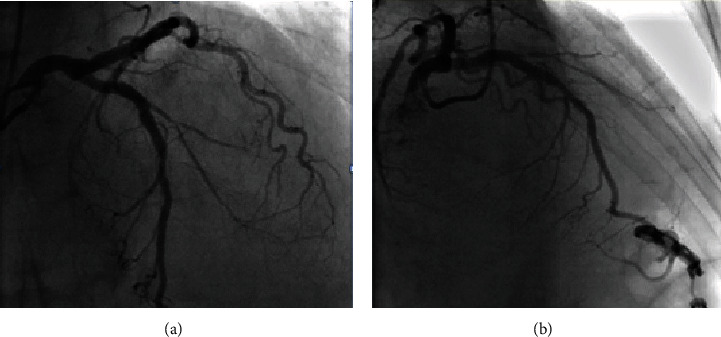
(a) AP cranial projection showing complete restoration of flow in the ruptured diagonal artery and no residual leakage after 48 hours. (b) LAO caudal projection showing complete restoration of flow in the ruptured diagonal artery and no residual leakage after 48 hours.

After 24 hours the pigtail was removed, the repeated echocardiography showed no pericardial effusion. Repeated angiography was done the next day which showed complete restoration of flow in the diagonal artery and no leakage (Video 12) (Figure 5). During the following three years after her procedure, she had no angina symptoms and is doing well.

## 3. Discussion

In this case, wire-induced coronary rupture happened during bifurcation stenting in a female patient with history of hypertension admitted for NSTEMI. Due to failed conservative management, the patient was treated with a novel product for sealing and treatment of the rupture. To our knowledge, this is the first case report using this material via intracoronary root for hemostatic purposes.

Our novel product “Spongostan” is an absorbable, sterile, water-insoluble, and malleable hemostatic gelatin sponge, intended for hemostatic use by applying to a bleeding surface.

This product is routinely used by ENT surgeons for hemostatic purposes [7]. It has also been used recently for endovascular hemostasis and controlling bleeding in thyroid surgeries [8, 9].

It has a couple of advantages over standard treatment. It is very inexpensive, very easy to use, needs a very short time to prepare, achieves complete sealing very fast, and most importantly, the flow will be restored after 4-6 hours completely after the ruptured vessel was sealed and will achieve complete sealing simply with increasing the injected material in a stepwise manner.

Although conservative management like wait and watch, prolonged balloon inflations using appropriate sized low-pressure balloon inflation, and reversal of heparin are among first steps, more than often more aggressive treatments like fat embolization, coiling, glue injection, or stent grafts are needed as standard treatment based on the situation and localization of the rupture. Each of these treatments has some disadvantages [6, 10]. Fat embolization is time-consuming to get ready, and coiling or glues are very expensive and are not readily available in many centers and are technically demanding to deploy. Stent grafts are expensive and have a very high rate of restenosis and poor long term results. Most importantly, all these methods will lose the affected artery for good and there will be no more flow in the affected vessel [6, 10].

## 4. Conclusions

Although coronary rupture can be a fatal complication, step-wise actions can help to overcome it. Having a high index of suspicion, timely diagnosis and management are important for managing such patients. The use of novel products can help us manage these patients with a better outcome.

## Figures and Tables

**Table 1:** Ellis classification.

Ellis classification	Description
Type I	Extraluminal crater without extravasation
Type II	Epicardial fat or myocardial blush without extravasation
Type III	Extravasation through a frank (>1 mm) perforation
Type IIICS	Extravasation through a frank (>1 mm) perforation into a circulatory chamber (e.g., left ventricle or coronary sinus)

## Data Availability

No datasets were generated or analyzed during the current study.

## Supplementary Materials

Supplementary Materials Video 1: coronary angiography showing significant stenosis in LAD artery and diagonal branch. Video 2: stent placed in LAD and side branch flow compromised. Video 3: rewiring of the diagonal branch. Video 4: when tip of the wire is causing rupture of the diagonal branch. Video 5: leakage in the diagonal branch. Video 6: using balloon inflation to seal the rupture. Video 7: rupture under control after prolonged balloon inflation. Video 8: pigtail insertion after 4 hours due to tamponade and angiography showing diagonal leakage. Video 9: using balloon inflation for sealing the diagonal branch. Video 10: injecting Spongostan using microcatheter. Video 11: sealed diagonal branch. Video 12: coronary angiography after 48 hours showing restored diagonal flow without leakage.
